# The Distribution of Sport Performance Gene Variations Through COVID-19 Disease Severity

**DOI:** 10.3390/diagnostics15060701

**Published:** 2025-03-12

**Authors:** Guven Yenmis, Ilayda Kallenci, Mehmet Dokur, Suna Koc, Sila Basak Yalinkilic, Evren Atak, Mahmut Demirbilek, Hulya Arkan

**Affiliations:** 1 Department of Medical Biology, Tayfur Ata Sokmen School of Medicine, Hatay Mustafa Kemal University, Hatay 31060, Turkey; 2 Department of Molecular Biology and Genetics, Faculty of Natural Sciences and Engineering, Biruni University, Istanbul 34015, Turkey; 190401026@st.biruni.edu.tr (I.K.); 170401005@st.biruni.edu.tr (S.B.Y.); 3 Department of Emergency Medicine, Faculty of Medicine, Bilecik Seyh Edebali University, Bilecik 11230, Turkey; drdokur@gmail.com; 4 Department of Anesthesia and Reanimation, School of Medicine, Biruni University, Istanbul 34015, Turkey; skoc@biruni.edu.tr; 5 Department of Bioinformatics and System Biology, Institute of Natural and Applied Sciences, Gebze Technical University, Kocaeli 41400, Turkey; evrenatk98@gmail.com; 6 Department of Emergency Medicine, School of Medicine, Biruni University, Istanbul 34015, Turkey; mdemirbilek@biruni.edu.tr; 7 Department of Biotechnology, Institute of Science, Yildiz Technical University, Istanbul 34210, Turkey; hulya.arkan@std.yildiz.edu.tr

**Keywords:** COVID-19, biomarkers, ACTN3, ACE, PPARGC1A, intensive care unit, disease severity

## Abstract

**Background/Objectives:** Since its emergence in 2020, researchers worldwide have been collaborating to better understand the SARS-CoV-2 disease’s pathophysiology. Disease severity can vary based on several factors, including comorbidities and genetic variations. Notably, recent studies have highlighted the role of genes associated with athletic performance, such as ACE, ACTN3, and PPARGC1A, in influencing muscle function, cardiovascular health, and the body’s metabolic response. Given that these genes also impact oxidative metabolism, inflammation, and respiratory efficiency, we hypothesized that they might play a critical role in the host’s response to SARS-CoV-2 infection. This study aimed to investigate the association between disease severity and genetic polymorphisms in these sport performance-related genes, specifically ACE rs4646994, ACTN3 rs1815739, and PPARGC1A rs8192678. **Methods:** A total of 422 COVID-19-positive patients were included in this study. The participants were divided into three groups: a severe group (77 patients) requiring intensive care unit (ICU) admission, a mild group (300 patients) exhibiting at least one symptom, and an asymptomatic control group. Genotyping was performed using restriction fragment length polymorphism PCR. **Results:** The D allele and DD genotype of ACE and the T allele and TT genotype of ACTN3 were found to confer protective effects against severe SARS-CoV-2 infection. Conversely, the PPARGC1A TC genotype and the ACE-PPARGC1A ins/ins + TC combined genotype were associated with increased disease severity (*p* < 0.05). **Conclusions:** Although vaccination has reduced the severity of SARS-CoV-2, the virus continues to impact human health. Inter-individual differences due to these genetic variations will broaden the horizon of knowledge on the pathophysiology of the disease.

## 1. Introduction

The coronavirus disease 2019 (COVID-19), induced by the severe acute respiratory syndrome virus (SARS-CoV-2), has unexpectedly shifted into a pandemic, with more than 776 million confirmed cases and above 7 million deaths worldwide as of 22 September 2024 [1].

SARS-CoV-2 can cause severe and potentially life-threatening infections, particularly in elderly individuals with significant underlying health conditions. The most frequently reported comorbidities among COVID-19 patients are coronary artery disease (CAD) (6–8%), diabetes mellitus (DM) (19%), and hypertension (HT) (27–30%) [2,3]. In addition to these comorbidities, COVID-19 may manifest with a variety of clinical symptoms, including fever [4], fatigue [5], and severe complications such as sepsis [6], pulmonary embolism (PE) [7], bronchopneumonia (BP) [8], and acute respiratory distress syndrome (ARDS) [9]. These complications can exacerbate the severity of the disease and contribute to the progression of critical illness. Therefore, identifying correlations between these predictive factors may help reveal additional risk indicators, aiding in the identification and management of high-risk populations [10].

Moderate symptoms of COVID-19 may progress to fatal respiratory failure due to the development of ARDS. The renin–angiotensin–aldosterone system (RAAS) has been implicated in the pathophysiology of COVID-19 [11]. Additionally, the roles of angiotensin-converting enzymes 1 and 2 (ACE and ACE2) in ARDS progression are well established. Alveolar epithelial cells express high levels of ACE and ACE2, which serve as key regulators of RAAS by maintaining immunological and pulmonary vascular homeostasis. SARS-CoV-2 enters alveolar epithelial cells via membrane-bound ACE2 receptors. Disruption of the ACE/ACE2 counter-regulation through this mechanism leads to endothelial dysfunction and triggers severe, detrimental immune responses [12]. Common genetic variants in ACE genes have been associated with increased risk for HT, PE, renal failure, and cardiac diseases. Specifically, the 287 base pair ACE I/D polymorphism (rs4646994) is linked to elevated levels of ACE in the blood, an enzyme that counteracts the function of ACE2. The D/D genotype is reported to exhibit the highest ACE levels compared to the ID and II genotypes [13]. Due to the antagonistic relationship between ACE and ACE2, increased ACE expression is associated with reduced ACE2 receptor expression, and this polymorphism has been connected to poor outcomes in both ARDS and SARS [14]. Consequently, the presence of the D allele may also influence the clinical course of COVID-19 by lowering ACE2 receptor levels.

Alpha-actinin-3 (ACTN3) is a key component of the skeletal muscle Z-disk, specifically in fast-twitch muscle fibers [15], and interacts with various structural, signaling, and metabolic proteins [16,17]. A C>T substitution at codon 577 of the ACTN3 gene is a common genetic mutation, leading to a stop codon (X) and the production of a truncated protein [18]. The ACTN3 R577X polymorphism (rs1815739) results in ACTN3 deficiency, which is associated with reduced rapid contractile capacity and lower bone mass or density but enhanced endurance performance. This deficiency affects approximately 1.5 billion people worldwide, resulting in muscle weakness and a shift toward a more oxidative metabolism. In a comprehensive meta-analysis of the ACTN3 rs1815739 polymorphism, Alfred et al. demonstrated that the homozygous CC genotype is more common in Europeans, although it is not associated with athletic performance in the general population, whereas the homozygous TT genotype is frequently linked to improved aerobic fitness [19]. Some studies suggest that ACTN3 polymorphisms may influence the inflammatory response, which plays a critical role in determining COVID-19 severity [20]. Since fast-twitch fibers are also present in respiratory muscles, variations in ACTN3 could affect the strength and endurance of these muscles, potentially influencing respiratory efficiency and contributing to the severity of COVID-19 [21].

The PPARGC1A gene, located at the chromosomal position 4p15.2, plays key roles in fiber type determination, lipid metabolism, skeletal muscle fiber formation, and glucose regulation [22,23]. It encodes the transcriptional coactivator peroxisome proliferator-activated receptor gamma coactivator 1 alpha (PPARGC1A), which belongs to the peroxisome proliferator-activated receptor family. PPARGC1A activates various transcription factors that regulate a wide range of biological processes. Several amino acid polymorphisms have been identified within the coding region of PPARGC1A, including rs8192678 (Gly482Ser), which has been suggested to have functional significance [24]. The PPARGC1A rs8192678 polymorphism has been associated with high blood pressure, obesity, and DM [25,26,27]. Additionally, PPARGC1A plays a role in oxidative phosphorylation, which is essential for aerobic capacity and endurance [28]. As a result, polymorphisms in PPARGC1A have been linked to cardiovascular health [29]. Given the frequent cardiac complications observed in severe COVID-19 cases, these polymorphisms may be correlated with disease outcomes. Furthermore, PPARGC1A has been implicated in the regulation of inflammatory processes, mitochondrial efficiency, and energy production. Thus, variants in PPARGC1A could impact overall physical endurance and the body’s ability to mount an effective immune response to SARS-CoV-2, influencing disease severity [24,30].

Based on this background, the present study is set to investigate the roles of the ACE rs4646994, ACTN3 rs1815739, and PPARGC1A rs8192678 polymorphisms in determining the severity of COVID-19 outcomes. By doing so, we aim to contribute to the growing body of evidence on host genetic factors shaping individual variability in disease outcomes. We hypothesize that specific genotypes of these genes may confer protective or risk-enhancing effects, potentially explaining some of the inter-individual variability observed in COVID-19 severity. This research not only seeks to elucidate the genetic factors influencing COVID-19 outcomes but also explores the potential application of these findings in athletic populations to predict susceptibility to infectious diseases, such as COVID-19.

## 2. Materials and Methods

### 2.1. Samples

A total of 422 COVID-19-positive samples were collected from the patients who were admitted to Biruni University Hospital between March and October 2021. COVID-19 positivity was confirmed using RT-PCR assays conducted at Biruni University Hospital following WHO diagnostic protocols. The samples were divided into two main groups: COVID-19-positive asymptomatic control (outpatients with no symptoms and no need for hospitalization.) and COVID-19-positive symptomatic patients (inpatients or outpatients with symptoms). The symptomatic group was further divided into two subclasses: mild (symptoms requiring hospitalization without intensive care) and severe (cases requiring intensive care, mechanical ventilation, or resulting in death). Severity classification was based on WHO guidelines and confirmed by reviewing medical records.

The control group had 50 samples (age 31 ± 1.6), the mild group had 300 samples (age 48 ± 1.1), and the severe group had 77 samples (age 63 ± 1.8). The gender distribution was nearly equal across groups, with no significant gender bias (49.6% female). Exclusion criteria included teenagers and elderly patients (under 20 or over 65 years old), pregnant or breastfeeding patients, those with weakened immune systems (e.g., receiving chemotherapy), and patients unable to follow up due to various reasons. The demographic evaluation of age, gender, CAD, DM, HT, and smoking status is shown in Table 1. All patients were vaccinated with Pfizer-BioNTech and followed up for eight months to monitor disease progression.

### 2.2. Genotyping

Four milliliters of peripheral blood were drawn from each participant, and DNA was isolated using the Promega Wizard Genomic DNA Purification Kit. The concentration and optical density (OD) of DNA were measured using a NanoDrop spectrophotometer (NanoPhotometer P300, Implen GmbH, Munich, Germany). Samples with concentrations between 40 and 60 ng/µL and OD values of 1.8 ± 0.1 were included.

Polymerase Chain Reaction (PCR) was performed for each polymorphism according to the manufacturer’s instructions (PCR Master Mix (2×), Thermofisher Scientific, Catalog #K0172, Waltham, MA, USA). First, the DNA was denaturated at 94 °C for 2 min. Then, it went through 35 cycles of thermal cycling, which included denaturation at 94 °C for 30 s, primer annealing at 59 °C for 30 s, and extension at 72 °C for 30 s. The protocol concluded with a final extension step at 72 °C for 3 min. The primers were sense-CTGTAAGCCACTGCTGGAGA and antisense-AAATGAAGGGACCCAAGTG for ACE rs4646994, sense-GTGTGGCTGGTACACTCTGTG and antisense-CTGTCTCGGGCTCATCTGTA for ACTN3 rs1815739, and sense-TGCTACCTGAGAGAGACTTTGG and antisense-TGGAATATGGTGATCGGGAACA for PPARGC1A rs8192678.

**Figure 1:**
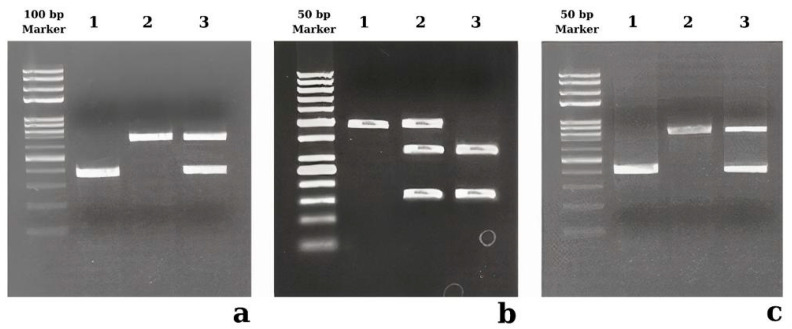
The restriction enzyme patterns of ACE rs4646994 (**a**), PPRGC1A rs8192678 (**b**), and ACTN3 rs1815739 (**c**) polymorphisms. (**a**) The first lane is a 100 bp size marker, lane 1 is del/del (homozygous), lane 2 is ins/ins (homozygous), and lane 3 is ins/del (heterozygous). (**b**) The first lane is a 50 bp size marker, lane 1 is TT (homozygous), lane 2 is TC (heterozygous), and lane 3 is CC (homozygous). (**c**) The first lane is a 50 bp size marker, lane 1 is TT (homozygous), lane 2 is TC (heterozygous), and lane 3 is CC (homozygous).

PCR products were electrophoresed at 120 V on a 2% agarose gel for 20 min using a Bio-Rad electrophoresis system (Sub-Cell Model 192 Cell, Biorad, Hercules, CA, USA), and the PCR fragments were monitored through a screening system (ChemiDoc MP, Biorad, Hercules, CA, USA). The PCR product of ACE rs4646994 has two alleles: 371 bp (the deletion allele) or 660 bp (the insert allele). The del/del genotype is observed as a single band with 371 bp in length, whereas the heterozygous del/ins genotype is observed as two distinct bands with 371 bp and 660 bp in length, and the ins/ins genotype is observed as a single band with 660 bp in length (Figure 1a).

The restriction fragment length polymorphism (RFLP)-PCR was used to find out the genotypes of the PPRGC1A rs8192678 and ACTN3 rs1815739 polymorphisms. One unit of the Mspl restriction enzyme (Catalog #R0106S, New England Biolabs, Ipswich, MA, USA) was used to digest the PPRGC1A PCR product. The PCR product–enzyme mixture was incubated at 37 °C for 15 min. The restriction-digested PCR product was electrophoresed at 100 V on a 2% agarose gel for 30 min. The homozygous rs8192678 CC genotype was observed as two bands of 149 bp and 311 bp in length; the heterozygous rs8192678 C/T genotype was observed as three distinct bands of 149 bp, 311 bp, and 460 bp in length; and the homozygous rs8192678 TT genotype was observed as a single band of 460 bp in length (Figure 1b).

One unit of the Ddel restriction enzyme (Catalog #R0175L, New England Biolabs, Ipswich, USA) was used to digest the ACTN3 PCR product. The PCR product and restriction enzyme mixture were incubated at 37 °C for 15 min, followed by thermal inactivation of the restriction enzyme at 65 °C for 15 min. The restriction-digested PCR product was electrophoresed on a 2% agarose gel at 100 V for 10 min. The homozygous rs1815739 CC genotype was observed as a single band of 412 bp in length, the heterozygous rs1815739 C/T genotype was observed as two distinct bands of 207 bp and 412 bp in length, and the homozygous rs1815739 TT genotype was observed as a single band of 207 bp in length (Figure 1c).

### 2.3. Statistical Analysis

Statistical analysis was conducted using GraphPad Prism 5.0 software (GraphPad Software, San Diego, CA, USA) and Minitab Statistical Software (Version 18.1) (Minitab Inc., State College, PA, USA). Sample size was determined based on power analysis (80% power, α = 0.05) to detect differences in genotype distributions between COVID-19 severity groups. The distribution of participants across groups was influenced by hospital admission records and clinical follow-ups. Age was displayed as mean ± standard error of the mean, demographic and symptomatic data were displayed as counts and percentages, and genotypes and allele frequencies were displayed as counts. To account for the age disparity between groups, statistical analyses controlled for age as a covariate in logistic regression models. The Mann–Whitney U test was used for the comparison of ages between the two groups. The Hardy–Weinberg equilibrium was determined for compatibility among groups using chi-square tests (χ^2^). The comparison of frequencies and ratios between groups was evaluated using chi-square tests or Fisher’s exact test. The associations between rs4646994, rs8192678, and rs1815739 genotypes, alleles, and COVID-19 were assessed using chi-square tests to calculate odds ratios (ORs), with homozygous dominant genotypes as the reference category for each polymorphism. A *p*-value of <0.05 was considered statistically significant.

## 3. Results

### 3.1. Demographic Evaluation of the Patients

A total of 377 COVID-19 patients were included in this study, comprising 300 mild cases with at least one symptom, 77 severe cases requiring intensive care, and 50 asymptomatic controls. The male percentage was 44% in the asymptomatic group, 50% in the mild group, and 55% in the severe group, with no significant gender distribution differences (*p* > 0.05). However, the mean age significantly increased with disease severity across all groups (*p* < 0.0001) (Table 2). In female individuals, age was significantly higher in the mild, severe, and overall groups compared to the control group (*p* < 0.0001 for all). Additionally, age was significantly higher in the severe group compared to the mild group (*p* < 0.0001).

Similarly, in male individuals, age was significantly higher in the mild (*p* = 0.0010), severe (*p* < 0.0001), and overall groups (*p* < 0.0001) compared to the control group. A significant increase in age was also observed in the severe group compared to the mild group (*p* = 0.0005) (Supplementary Files Tables S1 and S2).

Concerning the smoking history between the groups, 40% of the control group were active smokers, whereas the percentages were 43% in the mild and 68% in the severe groups. Smoking was found to be a 3.12-fold (*p* = 0.0022) and 2.741-fold (*p* = 0.0001) risk factor for the severe group compared to the asymptomatic control and the mild groups, respectively (Table 2). Moreover, no significant differences were found among female individuals (*p* > 0.05). However, in males, smoking was significantly associated with an increased risk in the severe group compared to the control group (*p* < 0.0001, OR = 20.00) and the overall group (*p* = 0.0285, OR = 2.642) (Supplementary Files Tables S1 and S2).

A total of 14% of the asymptomatic control group and 37% of all COVID-19 patients (31% mild and 61% severe) were found to have HT. There were no CAD patients in the asymptomatic control group; however, 12% of all COVID-19 patients were found to have CAD (9% mild and 25% severe). Compared to the control group, HT and CAD were found to be 3.603-fold (*p* = 0.0013) and 14.21-fold (*p* = 0.0088) risk factors in all COVID-19 patients. Similarly, HT and CAD were found to be 2.73-fold (*p* = 0.0149) and 10.19-fold (*p* = 0.0205) risk factors in the mild group and 9.624-fold and 33.67-fold risk factors in the severe group compared to the asymptomatic control group (*p* < 0.0001 for both). In line with these results, HT and CAD were found to be 3.525-fold (*p* < 0.0001) and 3.3-fold (*p* < 0.0002) risk factors for the severe group compared to the mild group (Table 2).

While 12% of the asymptomatic control group have DM, only 6% of all COVID-19 patients have DM. DM did not differ significantly between the groups (*p* > 0.05) (Table 2). In addition, the percentage of other chronic diseases such as COPD, CKD, and CKD varied from 0 to 4%, but these diseases seemed to have no significant contribution to COVID-19 pathogenesis (*p* > 0.05).

Concerning the symptoms, the frequency of fever was found to be 58% in all COVID-19 patients (72% in the mild group and 5% in the severe group). The presence of fever was found to increase the risk of disease 139.9 times in all COVID-19 patients and 254.6-fold in mild groups compared to the asymptomatic control group (*p* < 0.0001 for both) (Table 2).

Fatigue was observed in 58% of all COVID-19 patients. The presence of fatigue was found to increase the risk of disease 258.8-fold in mild groups and 139.9 times in all COVID-19 patients compared to the asymptomatic control group (*p* < 0.0001 for both). As the severe group was compared to the mild group, the presence of fever and fatigue was found to increase the severity of the disease from mild to severe by 0.022 and 0.016 folds, respectively (*p* < 0.0001) (Table 2).

The prevalence of sepsis in all COVID-19 patients was 27% (11% of the mild group and 90% of the severe group). The presence of sepsis was found to increase the overall risk of COVID-19 disease by 37.58 folds compared to the asymptomatic control group (*p* < 0.0001). Moreover, the presence of sepsis was found to increase the COVID-19 disease risk by 12.65 folds (*p* = 0.0076) in the mild group and by 825.8 folds (*p* < 0.0001) in the severe group, and this risk was shown to be 69.78 folds higher in the severe group than in the mild group (*p* < 0.0001) (Table 2).

The prevalence of ARDS in all COVID-19 patients was 23% (10% of the mild group and 73% of the severe group). The presence of ARDS was found to increase the risk of COVID-19 disease by 29.52 folds compared to the asymptomatic control group (*p* < 0.0002). Additionally, the presence of ARDS was found to increase the risk by 10.97 folds (*p* = 0.0219) in the mild group and 265.4 folds (*p* < 0.0001) in the severe group, and the risk was found to be 24.92 folds higher in the severe group than in the mild group (*p* < 0.0001) (Table 2).

The prevalence of PE and BP were found to be 5% and 8% in all COVID-19 patients (5% and 9% of the mild group and 3% and 5% of the severe group, respectively). The presence of BP was found to increase the risk of COVID-19 disease in overall COVID-19 patients by 8.865 folds compared to the asymptomatic control group (*p* = 0.0362). Furthermore, the presence of BP is shown to increase the risk by 9.75 folds (*p* = 0.0354) in the mild group. There was no significant difference in the prevalence of PE and BP between the mild and severe groups (*p* = 0.5415 and *p* = 0.3153, respectively) (Table 2).

### 3.2. The Genotype Analysis of ACE1 rs4646994, PPARGC1A rs8192678, and ACTN3 rs1815739 Polymorphisms

The effects of ACE1 rs4646994, PPARGC1A rs8192678, and ACTN3 rs1815739 polymorphisms on COVID-19 severity were evaluated. All polymorphisms were in Hardy–Weinberg equilibrium (HWE). ACE1 rs4646994 was found to have a protective effect, especially in female patients, where the DD genotype showed a 3.095-fold protective effect compared to II + ID (*p* = 0.01).

### 3.3. The Genotype Analysis of the ACE1 rs4646994 Polymorphism

A significant protective effect was observed for the ACE1 rs4646994 DD genotype in the severe group compared to the mild group, with a 1.912 times protective role (*p* = 0.0185) (Table 3). In females, the protective role of the DD genotype was even higher, with a 3.095-fold protective effect (*p* = 0.01). Additionally, the ID genotype was associated with a 1.937 times increased risk of severe disease in females (*p* = 0.0112) (Supplementary Files Tables S3 and S4).

### 3.4. The Genotype Analysis of the PPARGC1A rs8192678 Polymorphism

The PPARGC1A rs8192678 TC genotype was found to significantly increase the risk of COVID-19 disease by 4.122-fold in all patients (*p* < 0.0001), with similar increases observed in both the mild and severe groups. The T allele was found to increase disease risk only in the mild group by 1.565-fold (*p* = 0.0456). In the recessive model, TC + TT increased the COVID-19 disease risk by 3.054-fold in all COVID-19 patients, by 3.167-fold in the mild group, and by 2.667-fold in the severe group (*p* = 0.0002, *p* = 0.0001, and *p* = 0.0092, respectively) (Table 4).

As the polymorphism was analyzed in terms of gender, carrying the TC genotype increased the risk of COVID-19 disease by 4.625-fold in women (*p* = 0.0006) and 3.542-fold in men (*p* = 0.011) of overall COVID-19 patients, by 4.74-fold in women (*p* = 0.0006) and 3.651-fold (*p* = 0.0106) in men of the mild group, and by 4.167-fold (*p* = 0.0141) in women and 3.214-fold (*p* = 0.0481) in men of the severe group. In the recessive model, in women, TC + TT increased the risk of COVID-19 disease by 3.564-fold in overall COVID-19 patients, by 3.622-fold in the mild group, and by 3.333-fold in the severe group (*p* = 0.0014, *p* = 0.0016, and *p* = 0.0237). In men, however, TC + CC increased the risk of disease by 2.517-fold in overall COVID-19 patients and by 2.662-fold in the mild group (*p* = 0.0393 and *p* = 0.0321, respectively) (Supplementary Files Tables S5 and S6).

### 3.5. The Genotype Analysis of the ACTN3 rs1815739 Polymorphism

For ACTN3 rs1815739, the TT genotype was associated with an 8.389-fold reduction in COVID-19 disease risk (*p* < 0.0001) compared to the CC + TC genotypes. This protective effect was even more pronounced in the mild group, with a 16.15-fold reduction (*p* < 0.0001). In the gender-based analysis, the TT genotype was associated with a 9.308-fold reduction in disease risk in women and a 7.537-fold reduction in men (*p* < 0.0001).

When the effect of the T allele compared to the C allele on both overall COVID-19 patients and disease severity was examined, the T allele was associated with a 4.514-fold reduction in COVID-19 disease risk overall in COVID-19 patients and a 5.819-fold reduction in the risk in the mild group (*p* < 0.0001) (Table 5).

As women and men were evaluated separately, the T allele was associated with a 4.828-fold and 4.195-fold reduction in COVID-19 disease risk in overall COVID-19 patients (*p* = 0.0001 and *p* = 0.0015) and a 6.105-fold and 5.508-fold reduction in COVID-19 disease risk in the mild group (*p* < 0.0001 and *p* = 0.0001). Additionally, compared to the mild group, TT was associated with a 13.91-fold increase in COVID-19 disease risk in the severe group compared to CC + TC and a 5.11-fold increase in COVID-19 disease risk in the severe group compared to the C allele (*p* < 0.0001). Furthermore, compared to CC + TC, the TT genotype was associated with a 13.67-fold and 14.06-fold increase in COVID-19 disease risk in women and men in the severe group, respectively. The T allele was also associated with a 4.965-fold and 5.226-fold increase in COVID-19 disease risk in women and men of the severe group, respectively (*p* < 0.0001) (Supplementary File Tables S7 and S8).

### 3.6. Combined Genotype Analysis of ACE1 rs4646994, PPARGC1A rs8192678, and ACTN3 rs1815739 Polymorphisms

The combined analysis of ACE1 rs4646994 and PPARGC1A rs8192678 polymorphisms revealed significant interactions. For instance, the ins/ins + TC genotype was associated with a 7.6-fold increased risk of COVID-19 disease (*p* = 0.016) and a similar risk-8.25-fold increase in the severe group (*p* = 0.0393) (Table 6). No statistically significant differences were found when comparing the combined genotypes of ACE1 rs4646994 and ACTN3 rs1815739 (*p* > 0.05) (Table 7).

## 4. Discussion

The WHO database indicates that the susceptibility and severity of COVID-19 vary globally [1]. COVID-19 infection can result in a range of symptoms and complications that adversely affect athletic performance [31]. Some researchers have hypothesized that regional differences in gene frequencies may explain these variations [31,32]. Moreover, variations in the expression and function of immune response genes may underlie individual susceptibility to a disease, the risk of hospitalization, and the likelihood of adverse events.

Inherited and environmental factors that alter the expression and function of RAAS components, such as ACE, could explain the risk of developing COVID-19 and its adverse outcomes. Common variants in the two ACE genes have been linked to symptoms observed in COVID-19. In particular, the ACE rs4646994 polymorphism has been extensively studied due to its role in cardiovascular and pulmonary conditions [33,34]. The D allele of this polymorphism is associated with higher ACE expression, leading to reduced ACE2 receptor availability, the primary entry point for SARS-CoV-2 [14]. In accordance with these findings, in the present study, DD was found to have a 1.912 times more protective role compared to II + ID, and the D allele alone was found to have a protective role compared to the I allele. These findings support the hypothesis that ACE polymorphisms can modulate disease severity by affecting lung function and immune response during infection. However, some studies suggest that the D/D genotype might increase susceptibility to ARDS, a common complication in severe COVID-19 cases [13]. Therefore, it is hypothesized that having the D allele for the ACE I/D polymorphism may worsen the clinical course of COVID-19 by decreasing ACE2 receptor levels [35]. This suggests that individuals with the ACE DD genotype could benefit from targeted therapies aimed at modulating the RAS pathway, such as ACE inhibitors or angiotensin receptor blockers.

The PPARGC1A gene encodes a protein that regulates key genes involved in glucose and fatty acid metabolism [36,37]. The PPARGC1A rs8192678 polymorphism is also implicated in metabolic regulation, and polymorphisms in this gene have been linked to diabetes mellitus and hypertension, which are both risk factors for severe COVID-19 [25,26,27]. Physiological evidence suggests that this polymorphism affects blood lipid levels and insulin sensitivity, as the Ser allele carriers exhibit higher levels of insulin resistance and lipid dysregulation, which can lead to worse outcomes in patients already suffering from metabolic comorbidities [38,39]. As a consequence, these individuals have an elevated risk of type 2 DM [40,41]. Given that DM itself is a risk factor for severe COVID-19, the PPARGC1A rs8192678 polymorphism may influence COVID-19 pathophysiology. According to our results, the TC genotype was found to be a risk factor for all groups compared to the asymptomatic control group, and the T allele alone was found to be a risk factor compared to the C allele. This suggests that genetic variations in energy metabolism and oxidative phosphorylation, crucial during immune response, may determine how individuals respond to SARS-CoV-2 infection. Moreover, understanding this genotype’s role could pave the way for interventions, like antioxidant therapies or mitochondrial enhancers, to help reduce the severity of COVID-19 in affected individuals.

Several studies have examined the association between the PPARGC1A rs8192678 polymorphism and inflammation. One study identified a link between the A allele of this polymorphism and elevated levels of inflammatory markers, such as C-reactive protein (CRP), suggesting a possible connection between the A allele and heightened inflammatory responses [42]. Another study has demonstrated that the PPARGC1A rs8192678 risk A allele is associated with nonalcoholic fatty liver disease (NAFLD) and more severe forms of liver steatosis and activity grades in NAFLD patients, independent of PNPLA3 rs738409. This implies that the A allele may contribute to liver inflammation and disease progression [43]. Collectively, these findings suggest that the PPARGC1A rs8192678 polymorphism—particularly the A allele—is linked to increased inflammation and associated metabolic disturbances.

The ACTN3 R577X polymorphism, which affects about 1.5 billion people worldwide, results in a deficiency of the ACTN3 protein, a key component of fast-twitch muscle fibers. This deficiency leads to decreased muscle strength, improved endurance performance, and reduced bone mass. ACTN3 interacts with a variety of proteins involved in muscle structure, metabolism, and signaling, suggesting that its absence has a broad impact on muscle function [15,16,17]. The X allele of the ACTN3 gene, which is associated with less muscle mass, lower strength, and higher VO2 max, is more common in endurance athletes with a type I muscle fiber predominance. This suggests that the ACTN3 X allele may confer an advantage for endurance performance [44]. The XX genotype was also related to elevated cardiovascular fitness [45], which may explain the low VO2 max and high prevalence of CAD and HT in severe COVID-19 patients. This genotype results in reduced alpha-actinin-3, potentially impairing respiratory efficiency, which could worsen outcomes in patients with respiratory infections, like COVID-19. This genetic variation might be especially relevant for patients with pre-existing respiratory conditions or reduced muscle strength. The ACTN3 R577X polymorphism is a common nonsense mutation that results in the complete absence of ACTN3 in an estimated 16% of the global population [18]. Individuals with the RR genotype of the ACTN3 gene, which is associated with a higher proportion of type II muscle fibers and greater muscle strength, tend to perform better in strength and speed–power tests [46]. Interestingly, carriers of the ACTN3 X allele have a 1.72-fold higher risk of death than those with the ACTN3 577RR genotype in patients with congestive heart failure, suggesting that the ACTN3 genotype may be a prognostic marker for this condition [47]. This may be relevant to severe COVID-19 disease, as congestive heart failure can impair lung physiology. Emerging research has also examined the association between the ACTN3 rs1815739 polymorphism and lung health. A study by Shumna et al. (2020) [48] investigated this polymorphism in children with bronchial asthma, revealing that the C/C and C/T genotypes were correlated with impaired lung ventilation. Specifically, children carrying these genotypes exhibited lower forced vital capacity (FVC) and forced expiratory volume in one second (FEV_1_) compared to those with the T/T genotype, suggesting a potential relationship between the ACTN3 rs1815739 polymorphism and compromised pulmonary function in asthmatic children [48]. Another study sought to assess strength, respiratory muscle endurance, and lung function in patients with neuromuscular disorders (NMDs) who carry the ACTN3 R577X polymorphism. The findings indicated that the XX genotype might negatively impact respiratory muscle function in individuals with neuromuscular disorders [49]. The findings of the current study, by contrast, do not support the previous research. We found that carrying the ACTN3 577XX genotype or T allele alone may decrease the severity of the COVID-19 disease. The ACTN3 TT genotype appears to offer a protective effect, possibly by supporting better muscle function and reducing the likelihood of respiratory complications during severe illness. This subgroup may inherently face a lower risk of severe outcomes, providing valuable insights for resource allocation and tailored clinical management.

In terms of combined genetic effects, polymorphisms in ACE, ACTN3, and PPARGC1A likely interact to influence COVID-19 outcomes. The risk of COVID-19 disease was found to elevate in the presence of ACE1 rs4646994ins/ins + PPARGC1A rs8192678TC, ACE1 rs4646994ins/del + PPARGC1A rs8192678TC, and ACE1 rs4646994del/del+ PPARGC1A rs8192678TC genotypes, but no statistically significant differences were found when ACE1 rs4646994-ins/ins and ACTN3 rs1815739 CC combined genotypes compared to other genotypes. This suggests that genetic predispositions affecting muscle function, cardiovascular health, and metabolism collectively impact an individual’s risk of severe COVID-19.

## 5. Conclusions

To the best of our knowledge, this is the first study investigating the effect of ACTN3 and PPARGC1A polymorphisms on COVID-19 disease severity. The key limitation of case–control association studies is their small sample size, which can obscure true associations and lead to conflicting findings. Thus, the present findings in the Turkish population may require replication with larger sample sizes across multiple populations. Secondly, we did not follow the asymptomatic control study subjects after sample retrieval to determine if they developed disease symptoms. Third, this study did not include a non-infected control group, which would provide a stronger reference for assessing genotype effects. Future studies should incorporate non-infected individuals to better distinguish genetic risk factors for infection versus severity. Fourth, although all participants had received Pfizer-BioNTech vaccinations, we did not stratify disease severity outcomes based on vaccination status. Finally, this study did not assess participants’ physical activity levels, which could be a confounding factor, particularly in the context of ACTN3 and PPARGC1A polymorphisms.

This study underscores the potential impact of genetic screening in tailoring medical care and assessing COVID-19 risk. Identifying individuals with specific genetic variants linked to severe disease, such as the ACE DD or PPARGC1A TC genotypes, could enable healthcare providers to implement proactive monitoring, early intervention strategies, and lifestyle adjustments to reduce complications. Additionally, incorporating genetic markers like ACE rs4646994, ACTN3 rs1815739, and PPARGC1A rs8192678 into risk assessment models may improve patient triage, optimize resource allocation, and refine vaccination prioritization, particularly for vulnerable populations. These genetic insights could also shape personalized rehabilitation approaches, helping patients recover more effectively from severe COVID-19 outcomes.

Further research is essential to unravel how these polymorphisms influence COVID-19 progression and severity. Laboratory and animal studies could provide valuable insights into their effects on viral infection, immune response, and inflammation, potentially revealing new therapeutic targets. Additionally, large-scale genetic studies (GWASs) across diverse populations would help confirm these findings and expand their clinical relevance. Tracking patients with these genetic variants over time could shed light on disease progression and long-term effects. Furthermore, exploring how lifestyle factors such as exercise, nutrition, and medication interact with genetic predisposition could lead to preventive strategies that mitigate genetic risks, offering a more holistic approach to COVID-19 management.

## Figures and Tables

**Table 1:** Demographic evaluation of COVID-19 patients by the severity of COVID-19 disease.

	Control Group	Patient Groups		
	**Asymp.** **(*n* = 50)**	**Mild** **(*n* = 300)**	**Severe** **(*n* = 77)**	**Overall** **(*n* = 377)**
Age (M ± SEM)	31 ± 1.6	48 ± 1.1	63 ± 1.8	51 ± 1.0
Sex, *n* (%)				
Male	22 (44)	151 (50)	42 (55)	193 (51)
Female	28 (56)	149 (50)	35 (45)	184 (49)
Smoking, *n* (%)				
No	30 (60)	170 (57)	25 (32)	195 (52)
Yes	20 (40)	129 (43)	52 (68)	181 (48)
HT, *n* (%)				
No	43 (86)	207 (69)	30 (39)	237 (63)
Yes	7 (14)	92 (31)	47 (61)	139 (37)
DM, *n* (%)				
No	44 (88)	281 (94)	72 (94)	353 (94)
Yes	6 (12)	18 (6)	5 (6)	23 (6)
CAD, *n* (%)				
No	50 (100)	272 (91)	58 (75)	330 (88)
Yes	0 (0)	27 (9)	19 (25)	46 (12)
Fever, *n* (%)				
No	50 (100)	85 (28)	73 (95)	158 (42)
Yes	0 (0)	215 (72)	4 (5)	219 (58)
Fatigue, *n* (%)				
No	50 (100)	84 (28)	74 (96)	158 (42)
Yes	0 (0)	216 (72)	3 (4)	219 (58)
Sepsis, *n* (%)				
No	50 (100)	267 (89)	8 (10)	275 (73)
Yes	0 (0)	33 (11)	69 (90)	102 (27)
ARDS, *n* (%)				
No	50 (100)	271 (90)	21 (27)	292 (77)
Yes	0 (0)	29 (10)	56 (73)	85 (23)
PE, *n* (%)				
No	50 (100)	285 (95)	75 (97)	360 (95)
Yes	0 (0)	15 (5)	2 (3)	17 (5)
BP, *n* (%)				
No	50 (100)	274 (91)	73 (95)	347 (92)
Yes	0 (0)	26 (9)	4 (5)	30 (8)

One of the disease or smoking history data of a participant is missing in the mild group. M, mean; SEM, standard error of the mean; HT, hypertension; DM, diabetes mellitus; CAD, coronary artery disease; ARDS, acute respiratory distress syndrome; PE, pulmonary embolism; BP, bronchopneumonia.

**Table 2:** Comparative demographic evaluation of COVID-19 patients by the severity of COVID-19 disease.

	*p*-value (OR)			
	**Asymp. vs. Overall**	**Asymp. vs. Mild**	**Asymp. vs. Severe**	**Mild vs. Severe**
Age	<0.0001 *	<0.0001 *	<0.0001 *	<0.0001 *
Sex	0.3391 ^a^ (0.749)	0.4069 ^a^ (0.775)	0.2455 ^a^ (0.655)	0.5095 ^a^ (0.845)
Smoking	0.2788 ^a^ (1.392)	0.6774 ^a^ (1.138)	**0.0022** ^a^ (3.120)	**0.0001** ^a^ (2.741)
HT	**0.0013** ^a^ (3.603)	**0.0149** ^a^ (2.730)	**<0.0001** ^a^ (9.624)	**<0.0001** ^a^ (3.525)
DM	0.1328 ^b^ (0.478)	0.1318 ^b^ (0.470)	0.3398 ^b^ (0.509)	0.7950 ^b^ (1.084)
CAD	**0.0088** ^a^ (14.210)	**0.0205** ^b^ (10.190)	**0.0001** ^a^ (33.670)	**0.0002** ^a^ (3.300)
Fever	**<0.0001** ^a^ (139.9)	**<0.0001** ^a^ (254.6)	0.1532 ^b^ (6.184)	**<0.0001** ^a^ (0.022)
Fatigue	**<0.0001** ^a^ (139.9)	**<0.0001** ^a^ (258.8)	0.2782 ^b^ (4.745)	**<0.0001** ^a^ (0.016)
Sepsis	**<0.0001** ^a^ (37.58)	**0.0076** ^b^ (12.65)	**<0.0001** ^a^ (825.8)	**<0.0001** ^a^ (69.78)
ARDS	**0.0002** ^a^ (29.52)	**0.0219** ^b^ (10.97)	**<0.0001** ^a^ (265.4)	**<0.0001** ^a^ (24.92)
PE	0.2405 ^b^ (4.903)	0.1418 ^b^ (5.483)	0.5188 ^b^ (3.344)	0.5415 ^b^ (0.507)
BP	**0.0362** ^b^ (8.865)	**0.0354** ^b^ (9.750)	0.1532 ^b^ (6.184)	0.3153 ^a^ (0.577)

OR, odds ratio; M, mean; SEM, standard error of the mean; CAD, coronary artery disease; DM, diabetes mellitus; HT, hypertension. * Mann–Whitney U test, ^a^ the chi-square test or ^b^ Fisher’s exact test was used for analysis. *p* < 0.05 values are shown in bold.

**Table 3:** The distribution of the ACE1 rs4646994 polymorphism and disease severity.

**ACE1 rs4646994**	Control Group	Patient Groups			p-value (OR)			
**Genotypes**	**Asymp. (*n*)**	**Mild (*n*)**	**Severe (*n*)**	**Overall (*n*)**	**Asymp.** **vs.** **Overall**	**Asymp.** **vs.** **Mild**	**Asymp.** **vs.** **Severe**	**Mild** **vs.** **Severe**
II	8	48	15	63	Ref	Ref	Ref	Ref
ID	25	122	40	162	0.6517 (0.823)	0.6386 (0.813)	0.7541 (0.853)	0.8901 (1.049)
DD	17	130	22	152	0.7797 (1.135)	0.5980 (1.275)	0.4947 (0.690)	0.0988 (0.542)
Dominant Model (II + ID vs. DD)	33	170	55	225	Ref	Ref	Ref	Ref
	17	130	22	152	0.3906 (1.311)	0.2157 (1.484)	0.5170 (0.777)	**0.0185** (0.523)
Recessive Model (II vs. ID + DD)	8	48	15	63	Ref	Ref	Ref	Ref
	42	252	62	314	0.8991 (0.949)	1.0000 (1.000)	0.6188 (0.787)	0.4652 (0.787)
I	41	218	70	288	Ref	Ref	Ref	Ref
D	59	382	84	466	0.5883 (1.124)	0.3709 (1.218)	0.4844 (0.834)	**0.0377** (0.685)

Ref, reference; OR, odds ratio; a chi-square test was used for analysis. *p* < 0.05 values are shown in bold.

**Table 4:** The distribution of the PPARGC1A rs8192678 polymorphism and disease severity.

**PPARGC1A rs8192678**	Control Group	Patient Groups			p-Value (OR)			
**Genotypes**	**Asymp. (*n*)**	**Overall (*n*)**	**Mild (*n*)**	**Severe (*n*)**	**Asymp.** **vs.** **Overall**	**Asymp.** **vs.** **Mild**	**Asymp.** **vs.** **Severe**	**Mild** **vs.** **Severe**
CC	25	93	72	21	Ref	Ref	Ref	Ref
TC	15	230	183	47	**<0.0001** (4.122)	**<0.0001** (4.236)	**0.0013** (3.730)	0.6684 (0.881)
TT	10	54	45	9	0.3634 (1.452)	0.2854 (1.563)	0.8995 (1.071)	0.3911 (0.686)
Dominant model (CC + TC vs. TT)	40	323	255	68	Ref	Ref	Ref	Ref
	10	54	45	9	0.2907 (0.669)	0.3684 (0.706)	0.1995 (0.529)	0.4593 (0.750)
Recessive model (CC vs. TC + TT)	25	93	72	21	Ref	Ref	Ref	Ref
	25	284	228	56	**0.0002** (3.054)	**0.0001** (3.167)	**0.0092** (2.667)	0.5523 (0.842)
C allele	65	416	324	89	Ref	Ref	Ref	Ref
T allele	35	338	273	65	0.0626 (1.509)	**0.0456** (1.565)	0.2507 (1.356)	0.4336 (0.867)

Ref, reference; OR, odds ratio; the chi-square test was used for analysis. *p* < 0.05 values are shown in bold.

**Table 5:** The distribution of the ACTN3 rs1815739 polymorphism and disease severity.

**ACTN3 rs1815739**	Control Group	Patient Groups			p-value (OR)			
**Genotypes**	**Asymp. (*n*)**	**Overall (*n*)**	**Mild (*n*)**	**Severe (*n*)**	**Asymp.** **vs.** **Overall**	**Asymp.** **vs.** **Mild**	**Asymp.** **vs.** **Severe**	**Mild** **vs.** **Severe**
CC	0	5	5	0	Ref	Ref	Ref	Ref
TC	11	260	241	19	1.0000 ^b^ (2.059)	1.0000 ^b^ (1.909)	-	1.0000 ^b^ (0.888)
TT	39	112	54	58	0.3322 ^b^ (0.259)	0.1537 ^b^ (0.125)	-	0.0572 ^b^ (11.81)
Dominant model (CC + TC vs. TT)	11	265	246	19	Ref	Ref	Ref	Ref
	39	112	54	58	<0.0001 ^a^ (0.119)	<0.0001 ^a^ (0.062)	0.7288 ^a^ (0.861)	<0.0001 ^a^ (13.91)
Recessive model CC vs. TC + TT)	0	5	5	0	Ref	Ref	Ref	Ref
	50	372	295	77	1.0000 ^b^ (0.671)	1.0000 ^b^ (0.532)	-	0.5878 ^b^ (2.885)
C allele	11	270	251	19	Ref	Ref	Ref	Ref
T allele	89	484	349	135	**<0.0001** ^a^ (0.222)	**<0.0001** ^a^ (0.172)	0.7469 ^a^ (0.878)	**<0.0001** ^a^ (5.110)

Ref, reference; OR, odds ratio; ^a^ the chi-square test or ^b^ Fisher’s exact test was used for analysis. *p* < 0.05 values are shown in bold.

**Table 6:** The combined genotype analysis of the ACE1 rs4646994 and PPARGC1A rs8192678 polymorphisms.

**ACE1** **rs4646994** **+** **PPARGC1A rs8192678**	Control Group	Patient Groups			p-value (OR)			
	**Asymp. (*n*)**	**Overall (*n*)**	**Mild (*n*)**	**Severe (*n*)**	**Asymp.** **vs.** **Overall**	**Asymp.** **vs.** **Mild**	**Asymp.** **vs.** **Severe**	**Mild** **vs.** **Severe**
II + CC	6	15	11	4	Ref	Ref	Ref	Ref
II + TC	2	38	27	11	**0.0160** ^b^ (7.600)	**0.0380** ^b^ (7.364)	**0.0393** ^b^ (8.250)	1.0000 ^b^ (1.120)
II + TT	0	10	10	0	0.1411 ^b^ (8.806)	0.0570 ^b^ (11.87)	-	0.1245 ^b^ (0.122)
ID + CC	13	39	27	12	0.7529 ^a^ (1.200)	0.8378 ^a^ (1.133)	0.7233 ^b^ (1.385)	1.0000 ^b^ (1.222)
ID + TC	8	103	80	23	**0.0104** ^b^ (5.150)	**0.0102** ^b^ (5.455)	0.0645 ^b^ (4.313)	0.7449 ^b^ (0.791)
ID + TT	4	20	15	5	0.4764 ^b^ (2.000)	0.4629 ^b^ (2.045)	0.6563 ^b^ (1.875)	1.0000 ^b^ (0.917)
DD + CC	6	39	34	5	0.1749 ^b^ (2.600)	0.1522 ^b^ (3.091)	1.0000 ^b^ (1.250)	0.2436 ^b^ (0.404)
DD + TC	5	89	76	13	**0.0047** ^b^ (7.120)	**0.0031** ^b^ (8.291)	0.1245 ^b^ (3.900)	0.2628 ^b^ (0.470)
DD + TT	4	24	20	4	0.2906 ^b^ (2.400)	0.2697 ^b^ (2.727)	1.0000 ^b^ (1.500)	0.6857 ^b^ (0.550)

OR, odds ratio; Ref, reference; ^a^ the chi-square test or ^b^ Fisher’s exact test was used for analysis. *p* < 0.05 values are shown in bold.

**Table 7:** The combined genotype analysis of the ACE1 rs4646994 and ACTN3 rs1815739 polymorphisms.

**ACE1** **rs4646994** **+** **ACTN3** **rs1815739**	Control Group	Patient Groups			p-Value (OR)		
	**Asymp. (*n*)**	**Overall (*n*)**	**Mild (*n*)**	**Severe (*n*)**	**Asymp.** **vs.** **Overall**	**Asymp.** **vs.** **Mild**	**Mild** **vs.** **Severe**
II + CC	0	1	1	0	Ref	Ref	Ref
II + TC	0	43	42	1	-	-	1.0000 (0.106)
II + TT	8	19	5	14	1.0000 (0.765)	0.4286 (0.216)	0.3000 (7.909)
ID + TC	5	111	98	13	1.0000 (6.758)	1.0000 (5.970)	1.0000 (0.411)
ID + TT	20	50	23	27	1.0000 (0.821)	1.0000 (0.382)	0.4706 (3.511)
DD + TC	6	106	101	5	1.0000 (5.462)	1.0000 (5.205)	1.0000 (0.163)
DD + TT	11	43	26	17	1.0000 (1.261)	1.0000 (0.768)	1.0000 (1.981)

OR, odds ratio; Ref, reference; Fisher’s exact test was used for analysis. Since there was more than one 0 (zero) in the reference value, the asymptomatic control and severe groups could not be compared. In addition, ID + CC and DD + CC could not be compared due to the insufficient number of samples and were removed from the table.

## Data Availability

Data not included in the manuscript are shared as a separate Supplementary File.

## Supplementary Materials

The following supporting information can be downloaded at https://www.mdpi.com/article/10.3390/diagnostics15060701/s1, Table S1: Demographic evaluation of COVID-19 female patients by the severity of COVID-19 disease; Table S2: Demographic evaluation of COVID-19 male patients by the severity of COVID-19 disease; Table S3: The genotype–allele distribution of ACE1 rs4646994 polymorphism and disease severity in male participants; Table S4: The genotype–allele distribution of ACE1 rs4646994 polymorphism and disease severity in female participants; Table S5: The genotype–allele distribution of PPARGC1A rs8192678 polymorphism and disease severity in female participants; Table S6: The genotype–allele distribution of PPARGC1A rs8192678 polymorphism and disease severity in male participants; Table S7: The genotype–allele distribution of ACTN3 rs1815739 polymorphism and disease severity in female participants; Table S8: The genotype–allele distribution of ACTN3 rs1815739 polymorphism and disease severity in male participants.
