# Correction: Yenmis et al. The Distribution of Sport Performance Gene Variations Through COVID-19 Disease Severity. *Diagnostics* 2025, *15*, 701

**DOI:** 10.3390/diagnostics16142208

**Published:** 2026-07-15

**Authors:** Guven Yenmis, Ilayda Kallenci, Mehmet Dokur, Suna Koc, Sila Basak Yalinkilic, Evren Atak, Mahmut Demirbilek, Hulya Arkan

**Affiliations:** 1 Department of Medical Biology, Tayfur Ata Sokmen School of Medicine, Hatay Mustafa Kemal University, Hatay 31060, Turkey; 2 Department of Molecular Biology and Genetics, Faculty of Natural Sciences and Engineering, Biruni University, Istanbul 34015, Turkey; 3 Department of Emergency Medicine, Faculty of Medicine, Bilecik Seyh Edebali University, Bilecik 11230, Turkey; drdokur@gmail.com; 4 Department of Anesthesia and Reanimation, School of Medicine, Biruni University, Istanbul 34015, Turkey; skoc@biruni.edu.tr; 5 Department of Bioinformatics and System Biology, Institute of Natural and Applied Sciences, Gebze Technical University, Kocaeli 41400, Turkey; 6 Department of Emergency Medicine, School of Medicine, Biruni University, Istanbul 34015, Turkey; mdemirbilek@biruni.edu.tr; 7 Department of Biotechnology, Institute of Science, Yildiz Technical University, Istanbul 34210, Turkey; hulya.arkan@std.yildiz.edu.tr

## Error in Figure

In the original publication [1], there was a mistake in Figure 1 as published. The corresponding image file for Figure 1c had been inadvertently saved under the label “a” on the storage device, leading to the incorrect image being incorporated into the manuscript during figure preparation. The corrected Figure 1 and figure legend appear below. The authors state that the scientific conclusions are unaffected. This correction was approved by the Academic Editor. The original publication has also been updated.

## Figures and Tables

**Figure 1:**
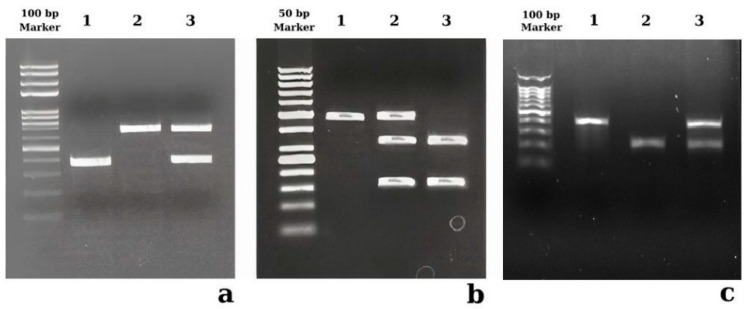
The restriction enzyme patterns of ACE rs4646994 (**a**), PPRGC1A rs8192678 (**b**), and ACTN3 rs1815739 (**c**) polymorphisms. (**a**) The first lane is a 100 bp size marker, lane 1 is del/del (homozygous), lane 2 is ins/ins (homozygous), and lane 3 is ins/del (heterozygous). (**b**) The first lane is a 50 bp size marker, lane 1 is TT (homozygous), lane 2 is TC (heterozygous), and lane 3 is CC (homozygous). (**c**) The first lane is a 100 bp size marker, lane 1 is CC (homozygous), lane 2 is TT (homozygous), and lane 3 is CT (heterozygous).
