# Supplementary material for: The Distribution of Sport Performance Gene Variations Through COVID-19 Disease Severity
Source: Diagnostics (Basel). 2025 Mar 12;15(6):701. doi: 10.3390/diagnostics15060701 (PMC11941099; doi:10.3390/diagnostics15060701)
Supplement: Supplementary file 1 [file diagnostics-15-00701-s001.zip › diagnostics-3438078-supplementary.pdf]

Supplementary Fıles

The Distribution of Sport Performance Gene Variations Through COVID-19 Disease Severity

Guven Yenmis [1,*], Ilayda Kallenci [2], Mehmet Dokur [3], Suna Koc [4], Sila Basak Yalinkilic [2], Evren Atak [5], Mahmut Demirbilek [6] and Hulya Arkan [7]

1 Department of Medical Biology, Tayfur Ata Sokmen School of Medicine, Hatay Mustafa Kemal University, Hatay 31060, Turkey
2 Department of Molecular Biology and Genetics, Faculty of Natural Sciences and Engineering, Biruni University, Istanbul 34015, Turkey; 190401026@st.biruni.edu.tr (I.K.); 170401005@st.biruni.edu.tr (S.B.Y.)
3 Department of Emergency Medicine, Faculty of Medicine, Bilecik Seyh Edebali University, Bilecik 11230, Turkey; drdokur@gmail.com
4 Department of Anesthesia and Reanimation, School of Medicine, Biruni University, Istanbul 34015, Turkey; skoc@biruni.edu.tr
5 Department of Bioinformatics and System Biology, Institute of Natural and Applied Sciences, Gebze Technical University, Kocaeli 41400, Turkey; evrenatk98@gmail.com
6 Department of Emergency Medicine, School of Medicine, Biruni University, Istanbul 34015, Turkey; mdemirbilek@biruni.edu.tr
7 Department of Biotechnology, Institute of Science, Yildiz Technical University, Istanbul 34210, Turkey; hulya.arkan@std.yildiz.edu.tr
* Correspondence: guven.yenmis@yahoo.com; Tel.: +90-535-787-7337

Table S1. Demographic evaluation of COVID-19 female patients by the severity of COVID-19 disease.

	Control Group	***Patient Groups***		
	**Asymp (n=28)**	**Mild (n=149)**	**Severe (n=35)**	**Overall (n=184)**
Age (M ± SEM)	28 ± 2.0	47 ± 1.6	67 ± 2.6	51 ± 1.5
Smoking, n (%)				
No	19 (68)	119 (80)	23 (66)	142 (78)
Yes	9 (32)	29 (20)	12 (34)	41 (22)
HT, n (%)				
No	26 (93)	101 (68)	10 (29)	111 (61)
Yes	2 (7)	47 (32)	25 (71)	72 (39)
DM, n (%)				
No	25 (89	141 (95)	32 (91)	173 (95)
Yes	3 (11)	7 (5)	3 (9)	10 (5)

CAD, n (%)				
No	28 (100)	142 (96)	24 (69)	166 (91)
Yes	0 (0)	6 (4)	11 (31)	17 (9)
Fever, n (%)				
No	28 (100)	36 (24)	34 (97)	70 (38)
Yes	0 (0)	113 (76)	1 (3)	114 (62)
Fatigue, n (%)				
No	28 (100)	36 (24)	34 (97)	70 (28)
Yes	0 (0)	113 (76)	1 (3)	114 (62)
Sepsis, n (%)				
No	28 (100)	138 (93)	5 (14)	143 (78)
Yes	0 (0)	11 (7)	30 (86)	41 (22)
ARDS, n (%)				
No	28 (100)	140 (94)	10 (29)	150 (82)
Yes	0 (0)	9 (6)	25 (71)	34 (18)
PE, n (%)				
No	28 (100)	143 (96)	35 (100)	178 (97)
Yes	0 (0)	6 (4)	0 (0)	6 (3)
BP, n (%)				
No	28 (100)	136 (91)	32 (91)	168 (91)
Yes	0 (0)	13 (9)	3 (9)	16 (9)

One of the disease or smoking history data of a participant is missing in the mild group. HT, Hypertension; DM, Diabetes Mellitus; CAD, Coronary Artery Disease; ARDS, Acute Respiratory Distress Syndrome; PE, Pulmonary Embolism; BP, Bronchopneumonia.

Table S2. Demographic evaluation of COVID-19 male patients by the severity of COVID-19 disease.

	Control Group	***Patient Groups***		
	**Asymp (n=22)**	**Mild (n=151)**	**Severe (n=42)**	**Overall (n=193)**
Age (M ± SEM)	35 ± 2.6	49 ± 1.6	59 ± 2.3	51 ± 1.4
Smoking, n (%)				

No	11 (50)	51 (34)	2 (5)	53 (27)
Yes	11 (50)	100 (66)	40 (95)	140 (73)
HT, n (%)				
No	17 ()	106 (70)	20 (48)	126 (65)
Yes	5 ()	45 (30)	22 (52)	67 (35)
DM, n (%)				
No	19 ()	140 (93)	40 (95)	180 (93)
Yes	3 ()	11 (7)	2 (5)	13 (7)
CAD, n (%)				
No	22 (100)	130 (86)	34 (81)	164 (85)
Yes	0 (0)	21 (14)	8 (19)	29 (15)
Fever, n (%)				
No	22 (100)	49 (32)	39 (93)	88 (46)
Yes	0 (0)	102 (68)	3 (7)	105 (54)
Fatigue, n (%)				
No	22 (100)	48 (32)	40 (95)	88 (46)
Yes	0 (0)	103 (68)	2 (5)	105 (54)
Sepsis, n (%)				
No	22 (100)	129 (85)	3 (7)	132 (68)
Yes	0 (0)	22 (15)	39 (93)	61 (32)
ARDS, n (%)				
No	22 (100)	131 (87)	11 (26)	142 (74)
Yes	0 (0)	20 (13)	31 (74)	51 (26)
PE, n (%)				
No	22 (100)	142 (94)	40 (95)	182 (94)
Yes	0 (0)	9 (6)	2 (5)	11 (6)
BP, n (%)				
No	22 (100)	138 (91)	41 (98)	179 (93)
Yes	0 (0)	13 (9)	1 (2)	14 (7)

HT, Hypertension; DM, Diabetes Mellitus; CAD, Coronary Artery Disease; ARDS, Acute Respiratory Distress Syndrome; PE, Pulmonary Embolism; BP, Bronchopneumonia.

Table S3 The genotype-allele distribution of ACE1 rs4646994 polymorphism and the disease severity in male participants

**ACE1** *rs4646994*	**Male**							
	Control	***Patients***			***p-value (OR)***			
**Genotypes**	**Asymp n**	**Overall n**	**Mild n**	**Severe n**	**Asymp vs Patient**	**Asymp vs Mild**	**Asymp vs Severe**	**Mild vs Severe**
Ins/Ins (II)	3	29	22	7	Ref	Ref	Ref	Ref
Ins/Del (ID)	11	84	64	20	1.0000[b] (0.790)	1.0000[b] (0.793)	1.0000[b] (0.779)	0.9715[a] (0.982)
Del/Del (DD)	8	80	65	15	1.0000[b] (1.034)	1.0000[b] (1.108)	1.0000[b] (0.804)	0.5357[a] (0.725)
Dominant Model (II+ID vs DD)	14	113	86	27	Ref	Ref	Ref	Ref
	8	80	65	15	0.6457[a] (1.239)	0.5532[a] (1.323)	0.9590[a] (0.972)	0.3936[a] (0.735)
Recessive Model (II vs ID+DD)	3	29	22	7	Ref	Ref	Ref	Ref
	19	164	129	35	1.0000[b] (0.893)	1.0000[b] (0.926)	1.0000[b] (0.790)	0.7365[a] (0.853)
Ins	17	142	108	34	Ref	Ref	Ref	Ref
Del	27	244	194	50	0.8098[a] (1.082)	0.7107[a] (1.131)	0.8400[a] (0.926)	0.4280[a] (0.819)

OR, odds ratio; Ref, reference; [a]Chi-square test or [b]Fisher's exact test were used for analysis.

Table S4 The genotype-allele distribution of ACE1 rs4646994 polymorphism and the disease severity in female participants

**ACE1** *rs4646994*	**Female**							
	Control	***Patients***			***p-value (OR)***			
**Genotypes**	**Asymp n**	**Overall n**	**Mild n**	**Severe n**	**Asymp vs Patient**	**Asymp vs Mild**	**Asymp vs Severe**	**Mild vs Severe**
Ins/Ins (II)	5	34	26	8	Ref	Ref	Ref	Ref
Ins/Del (ID)	14	78	58	20	0.7217[a] (0.819)	0.6907[a] (0.797)	0.8653[a] (0.893)	0.8124[a] (1.121)
Del/Del (DD)	9	72	65	7	0.7692[b] (1.176)	0.5488[b] (1.389)	0.3404[a] (0.486)	0.0747[b] (0.350)
Dominant Model (II+ID vs DD)	19	112	84	28	Ref	Ref	Ref	Ref
	9	72	65	7	0.4784[a] (1.357)	0.2584[a] (1.634)	0.2712[a] (0.528)	**0.0100**[a] (0.323)
Recessive Model (II vs ID+DD)	5	34	26	8	Ref	Ref	Ref	Ref
	23	150	123	27	0.9370[a] (0.959)	1.0000[b] (1.028)	0.6260[a] (0.734)	0.4583[a] (0.713)
Ins	24	146	110	36	Ref	Ref	Ref	Ref
Del	32	222	188	34	0.6507[a] (1.140)	0.4001[a] (1.282)	0.3384[a] (0.708)	**0.0255**[a] (0.553)

OR, odds ratio; Ref, reference; [a]Chi-square test or [b]Fisher's exact test were used for analysis. $p<0.05$ values were shown in bold

Table S5 The genotype-allele distribution of PPARGC1A rs8192678 polymorphism and the disease severity in female participants

**PPARGC1A *rs8192678***	**Female**							
	Control	***Patients***			***p-value (OR)***			
**Genotypes**	**Asymp n**	**Overall n**	**Mild n**	**Severe n**	**Asymp vs Patient**	**Asymp vs Mild**	**Asymp vs Severe**	**Mild vs Severe**
CC	15	45	36	9	Ref	**Ref**	**Ref**	Ref
TC	8	111	91	20	**0.0006**[a] (4.625)	**0.0006**[a] (4.740)	**0.0141**[a] (4.167)	0.7731[a] (0.879)
TT	5	28	22	6	0.2687[a] (1.867)	0.2945[a] (1.833)	0.4674[b] (2.000)	0.8832[a] (1.091)
Dominant Model (CC+TC vs TT)	23	156	127	29	Ref	Ref	Ref	Ref
	5	28	22	6	0.7794[b] (0.826)	0.7741[b] (0.797)	1.0000[b] (0.952)	0.7245[a] (1.194)
Recessive Model (CC vs TC+TT)	15	45	36	9	Ref	Ref	Ref	Ref
	13	139	113	26	**0.0014**[a] (3.564)	**0.0016**[a] (3.622)	**0.0237**[a] (3.333)	0.8474[a] (0.920)
C allele	38	201	163	38	Ref	Ref	Ref	Ref
T allele	18	167	135	32	0.0628[a] (1.754)	0.0682[a] (1.748)	0.1218[a] (1.778)	0.9503[a] (1.017)

OR, odds ratio; Ref, reference; [a]Chi-square test or [b]Fisher's exact test were used for analysis. $p<0.05$ values were shown in bold

Table S6 The genotype-allele distribution of PPARGC1A rs8192678 polymorphism and the disease severity in male participants

**PPARGC1A *rs8192678***	**Male**							
	Control	***Patients***			***p-value (OR)***			
**Genotypes**	**Asymp n**	**Overall n**	**Mild n**	**Severe n**	**Asymp vs Patient**	**Asymp vs Mild**	**Asymp vs Severe**	**Mild vs Severe**
CC	10	48	36	12	Ref	**Ref**	**Ref**	Ref
TC	7	119	92	27	**0.0110**[a] (3.542)	**0.0106**[a] (3.651)	**0.0481**[a] (3.214)	0.7494[a] (0.880)
TT	5	26	23	3	0.8938[b] (1.083)	0.6870[a] (1.278)	0.6817[b] (0.500)	0.1691[a] (0.391)
Dominant Model (CC+TC vs TT)	17	167	128	39	Ref	Ref	Ref	Ref
	5	26	23	3	0.3310[b] (0.529)	0.3613[b] (0.611)	0.1112[b] (0.262)	0.1744[a] (0.428)
Recessive Model (CC vs TC+TT)	10	48	36	12	Ref	Ref	Ref	Ref
	12	145	115	30	**0.0393**[a] (2.517)	**0.0321**[a] (2.662)	0.1768[a] (2.083)	0.5305[a] (0.783)
C allele	27	215	164	51	Ref	Ref	Ref	Ref
T allele	17	171	138	33	0.4730[a] (1.263)	0.3790[a] (1.336)	0.9430[a] (1.028)	0.2955[a] (0.769)

OR, odds ratio; Ref, reference; [a]Chi-square test or [b]Fisher's exact test were used for analysis. $p<0.05$ values were shown in bold

Table S7 The genotype-allele distribution of ACTN3 rs1815739 polymorphism and the disease severity in female participants

**ACTN3** ***rs1815739***	**Female**							
	Control	***Patients***			***p-value (OR)***			
**Genotypes**	**Asymp n**	**Overall n**	**Mild n**	**Severe n**	**Asymp vs Patient**	**Asymp vs Mild**	**Asymp vs Severe**	**Mild vs Severe**
CC	0	3	3	0	Ref	Ref	Ref	Ref
TC	6	129	120	9	1.0000[b] (2.846)	1.0000[b] (2.648)	-	1.0000[b] (0.552)
TT	22	52	26	26	0.5534[b] (0.333)	0.2494[b] (0.168)	-	0.2384[b] (7.000)
Dominant Model (CC+TC vs TT)	6	132	123	9	Ref	Ref	Ref	Ref
	22	52	26	26	**<0.0001**[a] (0.107)	**<0.0001**[a] (0.058)	0.6915[a] (0.788)	**<0.0001**[a] (13.67)
Recessive Model CC vs TC+TT)	0	3	3	0	Ref	Ref	Ref	Ref
	28	181	146	35	1.0000[b] (0.910)	1.0000[b] (0.734)	-	1.0000[b] (1.696)
C allele	6	135	126	9	Ref	Ref	Ref	Ref
T allele	50	233	172	61	**0.0001**[a] (0.207)	**<0.0001**[a] (0.164)	0.7121[a] (0.813)	**<0.0001**[a] (4.965)

OR, odds ratio; Ref, reference; [a]Chi-square test or [b]Fisher's exact test were used for analysis. p<0.05 values were shown in bold

Table S8 The genotype-allele distribution of ACTN3 rs1815739 polymorphism and the disease severity in male participants

**ACTN3** ***rs1815739***	**Male**							
	Control	***Patients***			***p-value (OR)***			
**Genotypes**	**Asymp n**	**Overall n**	**Mild n**	**Severe n**	**Asymp vs Patient**	**Asymp vs Mild**	**Asymp vs Severe**	**Mild vs Severe**
CC	0	2	2	0	Ref	Ref	Ref	Ref
TC	5	131	121	10	1.0000 (4.782)	1.0000[b] (4.418)	-	1.0000[b] (0.432)
TT	17	60	28	32	1.0000[b] (0.691)	0.5282[b] (0.326)	-	0.2300[b] (5.702)
Dominant Model (CC+TC vs TT)	5	133	123	10	Ref	Ref	Ref	Ref
	17	60	28	32	**<0.0001**[a] (0.133)	**<0.0001**[a] (0.067)	0.9227[a] (0.941)	**<0.0001**[a] (14.06)
Recessive Model CC vs TC+TT)	0	2	2	0	Ref	Ref	Ref	Ref
	22	191	149	42	1.0000[b] (1.702)	1.0000[b] (1.329)	-	1.0000[b] (1.421)
C allele	5	135	125	10	Ref	Ref	Ref	Ref
T allele	39	251	177	74	**0.0015**[a] (0.238)	**0.0001**[a] (0.182)	0.9280[a] (0.949)	**<0.0001**[a] (5.226)

OR, odds ratio; Ref, reference; [a]Chi-square test or [b]Fisher's exact test were used for analysis. p<0.05 values were shown in bold
